# That Wicked Theft

**Published:** 1876-04

**Authors:** 


### *THAT WICKED THEFT.*

Just as we go to press we receive a copy of the Cincinnati *Daily Star*, containing an advertisement which covers precisely half a page of that paper, and which emanates from the "Volta Belt Company" of Cincinnati. It appears that the "Volta Belt Company" is troubled because in our issue for January, 1876, we characterized their shameless distortion of an editorial which we wrote in our number for April, 1875, as "A Wicked Theft," and that they now seek to escape the penalty always attaching to unfair conduct, by decrying the standing and influence of the Journal of Health. We do not care to allude to this Belt Company again, having already stated in substance that we were compelled to look with suspicion upon a concern which could so far subvert our meaning by the omission of important words when claiming to quote our language, as to make us appear to be bearing testimony to the value of the appliances which they manufacture. It matters very little what opinion these belt persons may hold of the Journal; it is of great importance to all right-minded men that our utterances should not be wrested from their plain intent, and made to seemingly foster and sustain that to us utterly unknown quantity, the Volta Belt Company of Cincinnati.

The trouble appears to be that when we discussed the subject of electrical action and its application to diseased conditions, in our April, 1875, issue, we spoke some commendatory words of the appliances made by Mr. E. J. Seibert of this city. These belts, and bands, and shoe-soles we had used in several cases, and from their use excellent results had followed. We believed we were doing our readers a real service in explaining the uses and advantages of these simple and inexpensive instruments, at a time when, as never before, the minds of the medical profession are actively engaged in discussing the value of the various methods of inducing the electrical phenomena, and employing the power of this subtle and mysterious agent in the healing processes. We have examined scores of electrical disks and chains and belts from Pulvermacher's down, and we are bound to declare that the same principle is involved in all of them. To claim that the chain of Pulvermacher embodies any principle or exhibits any power which Seibert's goods do not, is the sheerest folly. In this view we are abundantly sustained by many medical writers. That able medical publication, the "New York Physican and Pharmacist" in its issue of November last, speaks as follows: "The Pulvermacher chain which has received the distinguished approbation of Sir Charles Locock and other European scientists, is largely used in England, and, as the Armadillo performs the same functions and presents the same principles, only in a more convenient form, there is no reason why it should not attain the same celebrity here."

So far as we have examined and tested the electric appliances of many makers, we discover no difference in power or in results. Seibert's goods have some advantages; they are of very convenient shape, are easily applied, and comfortably worn, and are quite reasonable in price. We have seen a good many sufferers from painful neuralgic affections, and distressing nervous disorders, who assert that these articles of Seibert's—to which he has given the general name of "Voltaic Armadillo"—have rendered signal and lasting service. Physicians innumerable have employed them, and testify to their efficacy. They are probably as good as anything of the kind can be, and will do all that ought to be expected of such appliances.

These Cincinnati belt people take great pains, and occupy much space to show that when Hall's Journal says a good word for Seibert's goods, it does it in spite of the adverse opinions of writers in the *Popular Science Monthly*, and the *Philadelphia Medical and Surgical Reporter*. This might all be true, and yet we should not consider it our duty to adopt the conclusions of persons who have not, it may be, given the subject as careful investigation as it has received at our hands. We find in the *Popular Science Monthly* of March, 1875, page 639, an editorial admission that the allusions to the Armadillo in a previous issue were made without any direct knowledge of the appliance. This is, of course, a complete retraction of any previous criticism upon the instruments. The article in the *Philadelphia Medical and Surgical Reporter*, was published in the absence of the editor, who had never even seen the "Armadillo." It was prepared by a party in the interest of a rival house. This was the statement of the editor of the *Reporter* to another editor in New York. Of course, loose statements thus uttered will have little effect, when weighed with a mature judgment, based upon thoughtful, intelligent, and long-continued investigation.

But this unhappiness on the part of the Cincinnati belt personages, is a matter of no concern to the public. Suffering humanity only asks what it shall do to be saved from avoidable pain, and it is very likely to accept the answer which may be vouchsafed by the tried and reputable. We utter no "puffs;" the language of flattery is a dead tongue to us. We try to speak the truth, let it help or harm wheresoever it will. And with the simple thought uppermost in our minds that Mr. Seibert and his goods have been unjustly attacked, and that what we were impelled to say in their behalf has been employed, after distortion, to give credence and popularity to a device of which we know nothing, we are impelled to say that we have found the Voltaic Armadillo a valuable appliance in those cases in which a continuous electrical current has seemed to be indicated.

